# POPC Enhances Both the Maturation of Bovine Oocytes and the Subsequent Development and Quality of Embryos

**DOI:** 10.3390/ani15213172

**Published:** 2025-10-31

**Authors:** Xingyu Zhang, Daqing Wang, Xin Cheng, Yong Zhang, Ruizhen Jian, Jiajia Zhang, Guifang Cao

**Affiliations:** 1 College of Veterinary Medicine, Inner Mongolia Agricultural University, Hohhot 010011, China; 17725760839@163.com (X.Z.); wangdaqing050789@126.com (D.W.); 17822106812@163.com (X.C.); zhy1956@263.net (Y.Z.); jianruizhen0503@163.com (R.J.); 19193460688@163.com (J.Z.); 2 Animal Embryo and Developmental Engineering Key Laboratory of Higher Education, Institutions of Inner Mongolia Autonomous Region, Hohhot 010011, China; 3 Inner Mongolia Autonomous Region Key Laboratory of Basic Veterinary Medicine, Hohhot 010011, China; 4 Inner Mongolia Academy of Agricultural and Animal Husbandry Sciences, Hhhot 010031, China; 5 College of Life Sciences, Inner Mongolia University, Hohhot 010021, China

**Keywords:** in vitro maturation, oxidative stress, POPC, in vitro fertilization, reactive oxygen species, glutathione, mitochondrial

## Abstract

**Simple Summary:**

In vitro maturation (IVM) of oocytes is a critical step in livestock-assisted reproductive technologies. However, oxidative stress and mitochondrial dysfunction under in vitro conditions readily induce bovine oocyte aging, thereby limiting the efficiency of IVM. This study investigated the regulatory effects of palmitoyloleoylphosphatidylcholine (POPC) on IVM, oocyte aging, and developmental competence in bovine oocytes. Cumulus–oocyte complexes (COCs) were collected from slaughterhouse-derived ovaries and assigned to control (0 μmol/mL) and experimental groups supplemented with 50–200 μmol/mL of POPC. Following 22 h of culture at 38.5 °C under 5% CO_2_, the optimal concentration was determined through comprehensive analysis. Results demonstrated that 150 μmol/mL of POPC was the most effective treatment, significantly increasing the first polar body extrusion rate and cleavage rate of 2- to 4-cell embryos. This concentration effectively reduced reactive oxygen species (ROS) levels, elevated glutathione (GSH) content, and improved mitochondrial activity and spindle integrity. Furthermore, POPC modulated key molecular pathways by up-regulating SIRT1/2 and BCL-2 expression while down-regulating BAX and Caspase-1/3 activation, suggesting suppression of apoptosis and enhanced cellular resilience. These findings provide a mechanistic basis for optimizing livestock-assisted reproductive technologies through targeted lipid supplementation.

**Abstract:**

In vitro maturation (IVM) of oocytes is a pivotal step in assisted reproductive technologies for livestock. However, oxidative stress (OS) and mitochondrial dysfunction during in vitro culture often lead to oocyte aging, thereby limiting the efficiency of the technologies. To address these challenges, this study investigated the regulatory effects of 1-Palmitoyl-2-Oleoyl-sn-Glycero-3-Phosphocholine (POPC) on bovine oocyte IVM, aging, and developmental competence to determine the optimal concentration and explore underlying mechanisms. Cumulus–oocyte complexes (COCs) were collected from abattoir-derived bovine ovaries and cultured in IVM medium supplemented with 0 (control), 50, 100, 150, or 200 μmol/mL of POPC (*n* = 300 per group) at 38.5 °C under 5% CO_2_ for 22 h. The optimal concentration was determined based on the first polar body extrusion rate, followed by in vitro fertilization (IVF), fluorescence staining, Smart-seq2 transcriptome sequencing, and quantitative PCR (qPCR) analysis. The results demonstrated that 150 μmol/mL of POPC yielded the highest maturation rate, significantly exceeding the control group (*p* < 0.05), and enhanced 2-4-cell cleavage rates after IVF. Furthermore, POPC markedly reduced intracellular reactive oxygen species (ROS) levels, increased glutathione (GSH) content, improved mitochondrial function, and restored normal spindle morphology. Transcriptomic analysis identified 350 upregulated and 280 downregulated differentially expressed genes (DEGs), which were enriched in pathways related to OS. qPCR validation confirmed upregulation of SIRT1/2 and BCL-2, along with downregulation of BAX and Caspase-1/3. Collectively, these findings suggest that 150 μmol/mL of POPC alleviates OS and activates the “SIRT–antioxidant–antiapoptotic” signaling axis, thereby providing valuable insights for optimizing assisted reproductive technologies in livestock.

## 1. Introduction

As the fundamental material for gametogenesis and embryogenesis in livestock, oocytes play an irreplaceable strategic role in genetic improvement [1], productivity enhancement, and germplasm conservation. In vitro maturation (IVM) of oocytes constitutes a pivotal component of the assisted reproductive technology system in domestic animals [2]. The efficiency of IVM and the quality of matured oocytes directly determine the success of subsequent embryo engineering procedures, such as in vitro fertilization (IVF), somatic cell nuclear transfer (SCNT) [3,4], and embryo cryopreservation [5], providing the essential biological foundation for superior breeding and genetic improvement in animal husbandry. Given that oocyte quality is a decisive factor governing embryonic developmental competence, elucidating the molecular mechanisms regulating oocyte quality and establishing effective strategies for its enhancement are of both theoretical and practical importance for overcoming the current bottlenecks in livestock reproductive efficiency.

The maturation status of oocytes is a critical indicator of the success rate of IVF and SCNT. In practical applications, the widespread occurrence of oocyte aging during in vitro culture markedly compromises the efficiency of these techniques. This is primarily attributed to two aspects. First, intrinsic heterogeneity in genetic background and developmental stage leads to asynchronous progression during IVM. Second, procedural delays and environmental fluctuations—such as variations in temperature and pH during oocyte collection, washing, and medium transfer—further exacerbate oocyte aging. Oocyte aging exerts significant detrimental effects on multiple key parameters of assisted reproduction. Specifically, it reduces sperm–oocyte binding and fertilization rates during IVF, impairs donor cell–oocyte fusion efficiency in SCNT, and markedly decreases the cleavage rate, blastocyst formation rate, and cell viability of reconstructed embryos, ultimately resulting in declined pregnancy rates following embryo transfer [6].

With the deepening understanding of the molecular mechanisms underlying oocyte aging, identifying the key regulatory factors governing oocyte developmental competence and maturation quality has emerged as an urgent and central issue for advancing the translational application and efficiency of livestock embryo engineering technologies [7]. From a morphological and functional perspective, oocyte aging is accompanied by characteristic phenotypic alterations, which can serve as intuitive indicators of quality deterioration. These include a markedly widened perivitelline space, suggesting cytoplasmic contraction and compromised plasma membrane integrity; chromatin condensation and fragmentation within the first polar body, indicative of meiotic dysregulation; zona pellucida crosslinking and hardening, which increase sperm penetration resistance and directly impair fertilization; and abnormal spindle morphology characterized by disorganized microtubule alignment and polar body displacement [8], which compromises chromosomal segregation accuracy and elevates the risk of aneuploid embryo formation.

At the biochemical and molecular regulatory levels, disruption of intracellular redox homeostasis represents a hallmark of oocyte aging. This imbalance is primarily characterized by the pathological accumulation of reactive oxygen species (ROS) and the concurrent decline in the synthesis rate and increase in the consumption rate of glutathione (GSH)—a key component of the antioxidant defense system—resulting in a profound degradation in overall cellular antioxidant capacity [9,10]. As oxygen-containing reactive molecules with unpaired electrons, ROS exhibit a concentration-dependent dual role. Within physiological ranges, ROS function as signaling mediators involved in the initiation of oocyte meiotic resumption and in gap-junctional communication between cumulus cells and oocytes. Meanwhile, ROS modulate the expression of oxidative stress (OS)-related transcription factors, thereby participating in cell fate determination. However, when ROS production exceeds the neutralizing capacity of the antioxidant system, OS ensues. Excess ROS can attack the phospholipid bilayer of the plasma membrane, triggering lipid peroxidation and compromising membrane fluidity and integrity. Simultaneously, OS reduces the mitochondrial membrane potential (ΔΨm), inhibits the activity of mitochondrial respiratory chain complexes, and induces mitochondrial dysfunction, leading to diminished ATP production. Furthermore, OS can activate apoptosis-related proteases (such as caspase-3 and caspase-4) and initiate intrinsic apoptotic pathways [11], ultimately disrupting cellular homeostasis and causing oocyte functional decline.

Existing studies have suggested that the exogenous supplementation of phospholipid molecules may serve as a potential strategy to mitigate oocyte aging in vitro [12]. However, the underlying mechanisms and optimal application parameters remain to be fully elucidated. 1-Palmitoyl-2-Oleoyl-sn-Glycero-3-Phosphocholine (POPC), a principal constituent of the phospholipid bilayer, is abundantly distributed in tissues such as the brain, liver, kidney, and lungs. POPC molecules contain unsaturated fatty acid chains, which confer high membrane fluidity [13]. During the in vitro maturation (IVM) of bovine oocytes, exogenous supplementation of POPC—being one of the major phospholipid components of the cell membrane—helps stabilize oocyte membrane structure and reduce membrane damage caused by environmental fluctuations during in vitro manipulation and culture. POPC also exerts physiological regulatory functions by maintaining cell membrane integrity, participating in signal transduction pathways, and modulating energy metabolism. Evidence from previous studies indicates that POPC promotes the maturation of sheep oocytes [14].

In this study, different concentrations of POPC were supplemented into the IVM medium to investigate its regulatory effects on oocyte maturation, aging progression, and quality. The objectives were to identify the optimal concentration and time-dependent efficacy of POPC in alleviating oocyte aging in vitro, elucidate the molecular mechanisms by which phospholipids regulate oocyte quality, and provide both theoretical and experimental foundations for developing improved in vitro quality-enhancement systems for livestock oocytes.

## 2. Materials and Methods

### 2.1. Materials

Reagents: POPC (cat. no. 42773, Sigma-Aldrich, St. Louis, MO, USA), in vitro maturation medium (IVM Medium, cat. no. REF 1.03.020, Stroebech Medi, Hundested, Denmark), in vitro culture medium (IVC Medium, cat. no. REF 1.07.020, Stroebech Medi, Denmark), oocyte pickup medium (OPU Medium, cat. no. REF 2.01.500, Stroebech Medi, Denmark), ionomycin (cat. no. I3909, Sigma-Aldrich, Burlington, MA, USA), 6-(dimethylamino)purine (6-DMAP, cat. no. D2629, Sigma-Aldrich, USA), hyaluronidase (cat. no. H4272, Sigma-Aldrich, USA), Medium 199 (cat. no. 11150059, Gibco, Waltham, MA, USA), mineral oil (cat. no. M8410, Sigma-Aldrich, USA), Dulbecco’s phosphate-buffered saline (DPBS, cat. no. C3590-0500, Gibco, USA), penicillin-streptomycin solution (Double Antibiotics, cat. no. C3420-0100, Gibco, USA), reactive oxygen species (ROS) detection kit (cat. no. S0035S, Beyotime, China), CellTracker Blue CMF2HC Dye (cat. no. 215868-45-4, MedChemExpress, Monmouth Junction, NJ, USA), JC-1 staining solution (cat. no. C2003S, Beyotime, Shanghai, China), α-Tubulin (DM1A) mouse monoclonal antibody (mAb, cat. no. 3873T, Cell Signaling Technology, Danvers, MA, USA), goat anti-mouse IgG (H+L) cross-adsorbed secondary antibody, Alexa Fluor™ 488 (cat. no. A32723, Invitrogen, Waltham, MA, USA), MitoTracker Red CMXRos (cat. no. C1035, Beyotime, China), and DynaBeads mRNA Direct Kit (cat. no. 610.11, Thermo Fisher Scientific, Waltham, MA, USA) were purchased directly from the manufacturer’s official website and stored according to the manufacturers’ instructions.

Instruments: Electrophoresis system (Bio-Rad Laboratories, Hercules, CA, USA), pipettes (Eppendorf, Hamburg, Germany), CO_2_ incubator (Thermo Fisher Scientific, USA), benchtop centrifuge (Himac, Hitachi, Japan), refrigerated centrifuge (Thermo Fisher Scientific, USA), fluorescence microscopy imaging system (Olympus Corporation, Tokyo, Japan), and laser scanning confocal microscope (ZEISS LSM 880, Carl Zeiss AG, Oberkochen, Germany).

### 2.2. In Vitro Collection of Oocytes

Ovaries were collected from bovine sources at the Hohhot Beiya Slaughterhouse and transported to the laboratory within 2–3 h post-slaughter in pre-warmed physiological saline (DPBS) supplemented with gentamicin (50 μg/mL) to prevent microbial contamination. Upon arrival, ovaries were thoroughly rinsed three times with sterile phosphate-buffered saline (PBS) containing gentamicin until the wash solution was free of blood and debris. Follicular aspiration was performed using a sterile disposable 20 mL syringe fitted with an 18-gauge needle. All visible antral follicles (2–8 mm in diameter) on the ovarian surface were aspirated under laminar flow conditions. The aspirated follicular fluid was immediately transferred into a sterile Petri dish and allowed to settle briefly. Cumulus–oocyte complexes (COCs) were then identified and selected under a stereomicroscope based on morphological criteria: intact cytoplasm without signs of granulation or vacuolization, and complete enclosure by at least three layers of compact cumulus cells. Only high-quality COCs meeting these criteria were collected for subsequent in vitro maturation.

### 2.3. In Vitro Maturation (IVM) and In Vitro Fertilization (IVF)

In vitro maturation (IVM): Cumulus–oocyte complexes (COCs) surrounded by more than three layers of cumulus cells were washed three times sequentially with OPU medium, HM199, and IVM medium, respectively, and then transferred to 4-well culture plates (50 oocytes per 500 μL) containing IVM medium. The cultures were incubated in a CO_2_ incubator at 5% CO_2_, 38.5 °C, and maximum humidity for approximately 22 h. Maturation rate was determined as the percentage of oocytes that extruded the first polar body relative to the total number of collected oocytes.

In vitro fertilization (IVF): Frozen sperm samples were retrieved from liquid nitrogen storage (sperm preservation straws) provided by Inner Mongolia Sankexing Reproductive Biotechnology (Group) Co., Ltd. (Hohhot, China). The straws were gently flicked for 3–5 s, immersed in a 37 °C water bath for thawing, and dried with absorbent paper. The sealed end of each straw was cut off, and the contents were expelled into a 2 mL centrifuge tube containing culture medium by holding the straw vertically. Any residual semen was flushed out using a pipette. The sperm suspension was mixed with culture medium and centrifuged at 1500 rpm for 5 min. The supernatant was removed, with a small volume optionally retained to avoid pellet disturbance. The sperm pellet was resuspended in 1 mL of fresh culture medium and centrifuged again under the same conditions. After the second wash, the sperm were resuspended in IVF medium. If not used immediately, the suspension was kept upright at room temperature. Prior to use, the sample was gently pipetted to ensure uniform resuspension. The standard IVF insemination dose was 20 μL per drop, adjusted according to the number of drops required. Each fertilization drop contains 25 oocytes and a sperm concentration of 1 × 10^9^ viable spermatozoa per milliliter.

### 2.4. ROS Staining and GSH Staining

Mature denuded oocytes were subjected to reactive oxygen species (ROS) detection using the ROS assay kit. Briefly, oocytes were first washed once in phosphate-buffered saline (PBS), then transferred to a 5 μM solution of CM-H2DCFDA, and incubated at 37 °C for 30 min. Following incubation, the oocytes were washed three times with PBS. Green fluorescence was visualized and captured under a laser confocal microscope using an excitation wavelength of 495 nm and an emission wavelength of 530 nm.

For glutathione (GSH) detection, oocytes were stained with CellTracker Blue CMF2HC Dye. After one wash in PBS, oocytes were transferred to a 5 μM solution of CellTracker Blue CMF2HC Dye and incubated at 37 °C for 30 min. Subsequently, they were washed three times with PBS. Blue fluorescence was observed and recorded under a laser confocal microscope with an excitation wavelength of 450 nm and an emission wavelength of 460 nm.

### 2.5. Mitochondrial Membrane Potential Staining (MMP)

Mature denuded oocytes were subjected to mitochondrial membrane potential assessment using JC-1 staining. Briefly, oocytes were washed twice in phosphate-buffered saline (PBS), and the JC-1 staining solution was pre-warmed at 37 °C prior to use. Oocytes were then transferred to the JC-1 working solution and incubated in the dark at 37 °C for 20 min. After incubation, oocytes were washed three times with PBS. Fluorescence signals were visualized under a laser confocal microscope: JC-1 monomers exhibited green fluorescence with maximum excitation/emission wavelengths of 514 nm and 529 nm, respectively, while JC-1 aggregates (J-aggregates) emitted red fluorescence with maximum excitation and emission wavelengths of 585 nm and 590 nm. Both red and green fluorescence were captured for analysis.

### 2.6. Spindle Fiber Staining

For spindle microtubule immunofluorescence labeling, mature denuded oocytes were first washed twice in PBS and fixed in 4% paraformaldehyde for 20 min at room temperature. Following fixation, oocytes were permeabilized and blocked in a solution containing 0.5% Triton X-100 and 1% bovine serum albumin (BSA) for 30 min. After three washes in PBS (5 min each, totaling 15 min), oocytes were incubated overnight at 4 °C with primary antibody α-Tubulin (DM1A) Mouse mAb diluted in blocking buffer. The following day, oocytes were washed three times with PBS and subsequently incubated with secondary antibody—Goat anti-Mouse IgG (H + L) Cross-Adsorbed Secondary Antibody conjugated to Alexa Fluor™ 488—at 37 °C for 60 min. After another three PBS washes, oocytes were counterstained with DAPI solution at 37 °C for 15 min to label nuclei. Excess DAPI was removed by three additional PBS washes (5 min each). Finally, oocytes were mounted with an anti-fade mounting medium to prevent fluorescence quenching. Fluorescence imaging was performed using a laser confocal microscope: green fluorescence from Alexa Fluor™ 488 was detected at excitation/emission wavelengths of 499 nm/520 nm, and blue nuclear fluorescence from DAPI was observed at excitation/emission wavelengths of 364 nm/454 nm. Images of both channels were recorded simultaneously.

### 2.7. Mitochondrial Staining

Mitochondria were labeled using MitoTracker Red CMXRos. Mature denuded oocytes were washed twice in PBS, then transferred to a working solution of 5 μM MitoTracker Red CMXRos and incubated at 37 °C for 30 min in the dark. After incubation, oocytes were washed three times with PBS. Red fluorescence was visualized and captured under a laser confocal microscope using excitation and emission wavelengths of 579 nm and 599 nm, respectively.

### 2.8. Smart-Seq2 Single-Cell Sequencing Analysis

Following oocyte maturation, mature oocytes were individually collected from the blank control group and the 150 μmol/mL POPC treatment group for comparative analysis. For sample preparation, oocytes were first washed three times in HM199 medium with gentle pipetting to completely remove adherent cumulus cells and minimize contamination. Subsequently, oocytes were transferred to Ca^2+^ and Mg^2+^-free DPBS buffer and washed three additional times to further purify the samples. All washing procedures were performed on a pre-warmed heating block at 38.5 °C to maintain physiological conditions, and the entire process was completed within 10 min to ensure consistency and preserve oocyte viability. After washing, individual oocytes were carefully transferred into 0.2 mL RNase-free PCR tubes pre-loaded with 2.5 μL lysis buffer using micromanipulation techniques. The volume of carryover wash solution was strictly limited to ≤1 μL to prevent dilution of the lysis buffer and ensure efficient cell lysis and RNA release. Immediately after transfer, tubes were placed on ice to inhibit RNase activity, labeled clearly, sealed with adhesive film, and stored at −80 °C to maintain RNA integrity. To ensure reproducibility, this procedure was independently repeated across three biological replicates. All samples were subsequently shipped on dry ice to Lianchuan Biotechnology Co., Ltd. (Hangzhou, China) for Smart-seq2-based single-cell RNA sequencing.

### 2.9. Gene Expression Analysis of Bovine Oocytes

Marker genes were selected for quantitative analysis in the experiment, with GAPDH used as the internal reference gene. Total RNA was isolated from mature oocytes using the DynaBeads mRNA Direct Kit. Reverse transcription was performed using the same kit to generate cDNA. Briefly, 5 μL of RNA Annealing Buffer OT was added to 10 μL of extracted RNA, and the mixture was denatured at 65 °C for 3 min in a thermal cycler, followed by immediate cooling on ice. Then, 9 μL of cDNA Synthesis Buffer and 1 μL of a mixture containing RNA Script RT and RNase inhibitor were added. Reverse transcription was carried out at 37 °C for 50 min, followed by enzyme inactivation at 85 °C for 5 min, and then cooled to 4 °C prior to storage at −80 °C. For quantitative real-time PCR (qPCR), the reaction system (20 μL total volume) consisted of 1 μL cDNA template, 1 μL each of forward and reverse primers, 10 μL of qPCR Master Mix, and 7 μL of nuclease-free water. Amplification was performed under the following conditions: initial denaturation at 95 °C for 3 min, followed by 40 cycles of 95 °C for 15 s, 60 °C for 30 s, and 72 °C for 2 s, with a final extension at 72 °C for 5 min. Relative gene expression levels were calculated using the 2^−ΔΔCT^ method (Table 1).

## 3. Data Analysis

In this study, fluorescence intensity associated with gene expression was quantified using ImageJ 1.8.0 software (National Institutes of Health, Bethesda, MD, USA) to determine relative gene expression levels. To ensure result reliability and reproducibility, each experiment was independently repeated at least three times to provide adequate biological and technical replication. All statistical analyses were conducted using Prism 7 software (GraphPad Software, Inc., San Diego, CA, USA), and data are expressed as mean ± standard deviation (SD). Statistical significance was assessed by one-way analysis of variance (ANOVA) followed by Tukey’s multiple comparisons test or two-way ANOVA, as appropriate. *p*-value ≤ 0.05 was considered statistically significant, with significance levels denoted as follows: ns (not significant, *p* > 0.05), * (*p* < 0.05), ** (*p* < 0.01), *** (*p* < 0.001).

## 4. Results

### 4.1. Effects of Exogenous POPC on In Vitro Maturation and Embryonic Development Potential of Oocytes

To investigate the dose-dependent effects of exogenous POPC on in vitro oocyte maturation and subsequent embryonic development, this study established a control group with 0 μmol/mL of POPC and experimental groups supplemented with 50, 100, 150, or 200 μmol/mL of POPC. A total of 300 morphologically intact cumulus–oocyte complexes (COCs) were allocated to each group, and the experiment was independently repeated three times. Following standard in vitro culture for 22 h, the first polar body extrusion rate, cumulus cell expansion rate, and perivitelline space width were assessed and statistically analyzed. Subsequently, in vitro fertilization (IVF) was performed on oocytes from the group exhibiting optimal maturation outcomes to evaluate the impact of POPC on early embryonic development.

**Figure 1:**
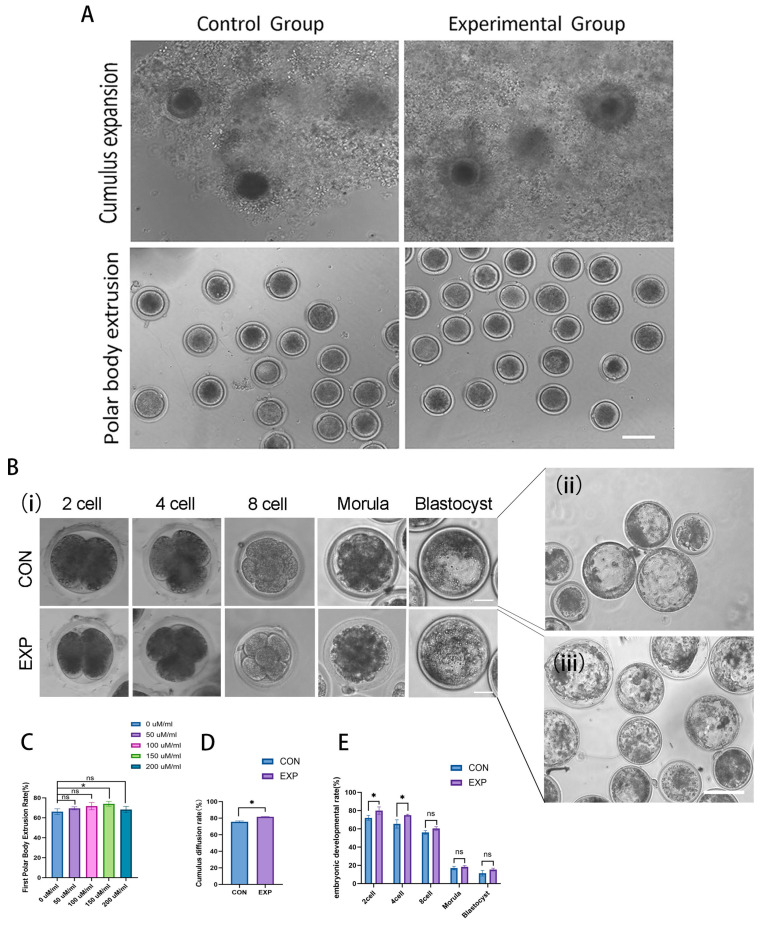
Effects of POPC on in vitro oocyte maturation and embryonic developmental potential. (**A**): Cumulus expansion and oocyte maturation morphology following supplementation with 150 μmol/mL of POPC (scale bar: 20 μm). (**B**): Morphological characteristics of early embryos at different developmental stages ((**i**): scale bar: 20 μm; (**ii**,**iii**): scale bar: 100 μm). (**C**): First polar body extrusion rates of oocytes matured under varying concentrations of POPC. (**D**): Comparison of cumulus expansion rates between experimental and control groups. (**E**): Embryo development rates across experimental and control groups (ns: not significant, *p* > 0.05, *: *p* < 0.05).

The results demonstrated that all POPC-supplemented groups supported oocyte maturation. Notably, the 150 μmol/mL group exhibited robust cumulus cell expansion and a perivitelline space width less than 3 μm. This group showed a significantly higher first polar body extrusion rate (76.0%) compared to the control group (*p* < 0.05), along with a significantly enhanced cumulus expansion rate (*p* < 0.05). In contrast, higher concentrations of POPC did not yield additional benefits (Figure 1A,C,D). In IVF experiments, embryos derived from the 150 μmol/mL group displayed normal morphology and developed successfully to the blastocyst stage. The cleavage rates at the 2-cell and 4-cell stages were significantly improved relative to the control group (*p* < 0.05), although no significant difference was observed in blastocyst formation rates (Figure 1B,E).

These findings indicate that 150 μmol/mL of POPC effectively promotes oocyte maturation and early embryonic cleavage but does not significantly influence blastocyst development. The potential impact of this treatment on embryo implantation warrants further investigation.

### 4.2. Molecular Mechanisms Underlying POPC-Mediated Regulation of Oocyte Maturation

To elucidate the molecular mechanisms by which POPC regulates in vitro oocyte maturation, we evaluated oxidative stress status, glutathione (GSH) levels, and mitochondrial function. Mature oocytes from the 150 μmol/mL POPC treatment group and the 0 μmol/mL control group were used. Intracellular reactive oxygen species (ROS) levels were measured using the fluorescent probe DCFH-DA, while mitochondrial abundance was assessed using a green fluorescent probe. GSH content was determined using the CMF2HC Dye probe, with fluorescence signals analyzed via blue channel detection; mitochondrial mass was further quantified using a red fluorescent probe specific for mitochondria.

**Figure 2:**
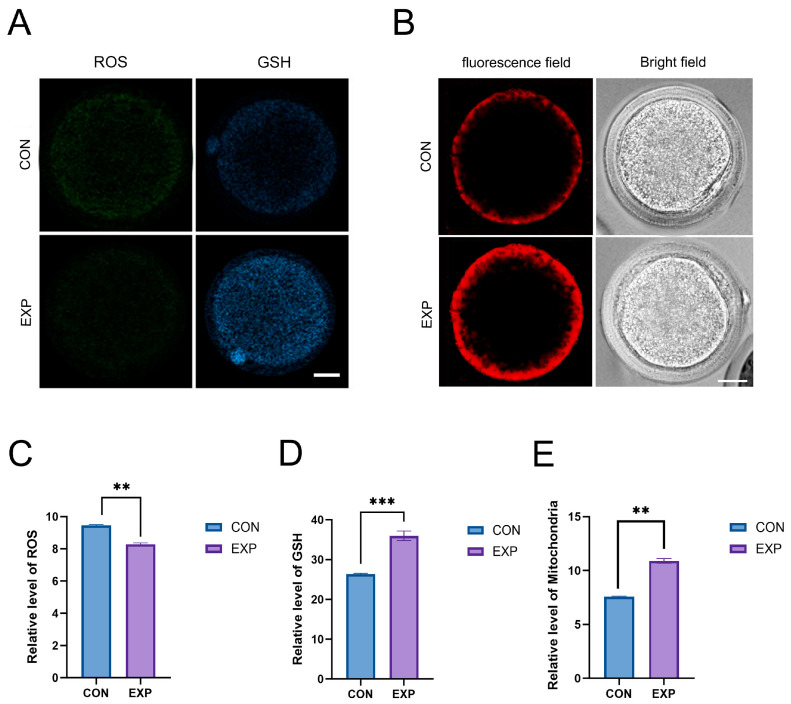
Molecular Mechanisms Underlying POPC-Mediated Regulation of Oocyte In Vitro Maturation. (**A**): Representative fluorescence images showing ROS and GSH levels in oocytes from the POPC-treated and control groups. (**B**): Representative images depicting mitochondrial structure in oocytes of both groups. (**C**): Normalized fluorescence intensity of ROS, showing a significant decrease in the POPC group compared to the control. (**D**): Normalized fluorescence intensity of GSH, which was significantly increased in the POPC group. (**E**): Normalized fluorescence intensity of mitochondria, indicating higher mitochondrial abundance in the POPC group (**: *p* < 0.01, ***: *p* < 0.001; Scale bar: 20 μm).

Results demonstrated that POPC significantly improved redox homeostasis in oocytes. Compared to the control group, oocytes treated with 150 μmol/mL of POPC exhibited a marked reduction in ROS levels (*p* < 0.01, Figure 2A,C) and a substantial increase in GSH content (*p* < 0.001, Figure 2A,D), indicating that POPC effectively mitigates oxidative stress by suppressing excessive ROS accumulation and enhancing antioxidant capacity through elevated GSH, thereby supporting intracellular stability.

Mitochondrial functional analysis revealed that oocytes in the 150 μmol/mL POPC group displayed significantly higher mitochondrial abundance compared to controls (*p* < 0.01, Figure 2B,E). This suggests that POPC enhances oocyte energy supply by increasing mitochondrial content, which is closely associated with its positive effects on oocyte maturation and early embryonic cleavage. These findings provide direct mitochondrial-level evidence for the role of POPC in regulating oocyte developmental competence.

### 4.3. Immunostaining Analysis of Oocyte Organelles

**Figure 3:**
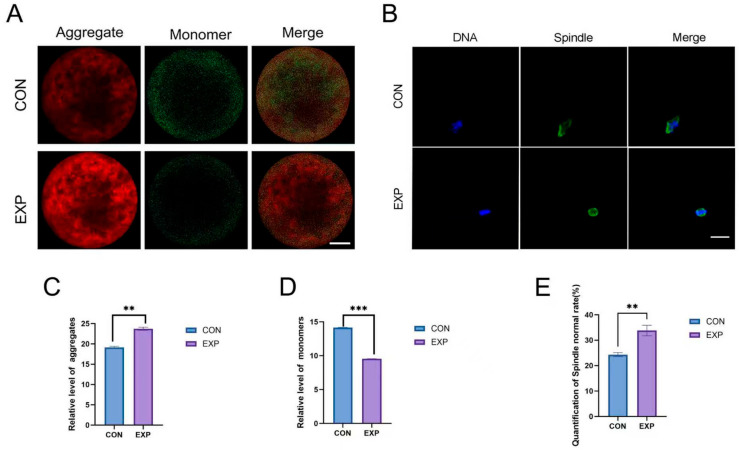
Immunofluorescence analysis of oocyte organelles. Panel (**A**): Representative images of mitochondrial membrane potential in oocytes (scale bar: 20 μm). Panel (**B**): Spindle structure visualized by α-tubulin immunostaining. Panel (**C**): Normalized fluorescence intensity of mitochondrial membrane potential aggregates, showing a significant increase in the POPC group. Panel (**D**): Normalized fluorescence intensity of monomeric (depolarized) mitochondrial membrane potential, which was markedly reduced. Panel (**E**): Quantitative assessment of normal spindle morphology, with a significantly higher proportion observed in the experimental group (**: *p* < 0.01, ***: *p* < 0.001; Scale bar: 20 μm).

Results demonstrated that POPC significantly enhanced mitochondrial function in oocytes. Compared with the control group, the experimental group exhibited a marked increase in the relative level of mitochondrial membrane potential aggregates (*p* < 0.01) and a pronounced reduction in depolarized monomers (*p* < 0.001) (Figure 3A,C,D), indicating that POPC helps maintain mitochondrial integrity by suppressing excessive depolarization. Spindle morphology analysis revealed that the proportion of oocytes with normal spindle configuration was significantly higher in the POPC group than in the control group (*p* < 0.01) (Figure 3B,E), confirming that POPC effectively preserves spindle polarity and structural integrity.

By protecting mitochondrial function and optimizing spindle architecture, POPC provides essential structural and energetic support for accurate chromosome segregation during meiosis, thereby uncovering a key mechanism through which it enhances oocyte meiotic competence.

### 4.4. Transcriptomic Profiling of Bovine MII Oocytes Treated with POPC Using Smart-Seq2

**Figure 4:**
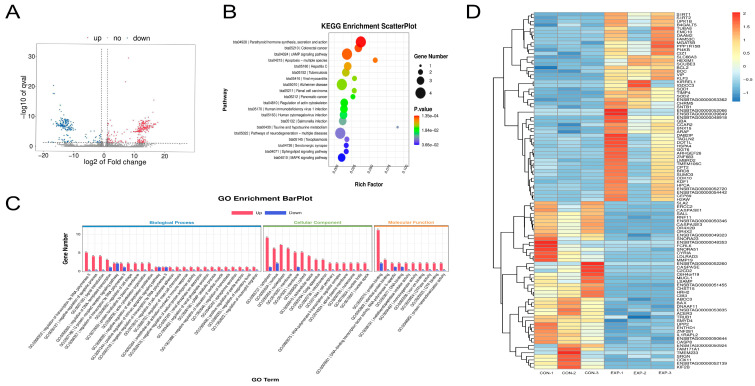
Transcriptomic analysis of oocytes using Smart-seq2. Panel (**A**): Volcano plot depicting differentially expressed genes (DEGs). The *x*-axis represents log_2_ fold change, indicating the magnitude of expression difference; the *y*-axis represents—log_10_ (*p* value), reflecting statistical significance. Red dots indicate upregulated DEGs, blue dots downregulated DEGs, and gray dots non-significant genes. Panel (**B**): KEGG pathway enrichment scatter plot. The *x*-axis shows the Rich Factor (number of DEGs enriched in a pathway divided by total gene number in that pathway), and the *y*-axis lists KEGG pathways. Dot color intensity (red to yellow) reflects increasing enrichment significance (lower *p*/*q* values), and dot size is proportional to the number of DEGs mapped to each pathway. Panel (**C**): GO enrichment bar chart categorized into three domains: Biological Process (BP), Cellular Component, and Molecular Function. The *x*-axis displays GO terms, and the *y*-axis indicates the number of associated DEGs. Red and blue bars represent upregulated and downregulated genes, respectively. Panel (**D**): Hierarchical clustering heatmap of DEG expression profiles. Rows represent individual DEGs, columns represent samples, with red and blue shades indicating high and low relative expression levels, respectively.

Transcriptome analysis revealed 350 significantly upregulated and 280 downregulated DEGs in the 150 μmol/mL POPC group compared to the control (Figure 4A). Functional enrichment analysis demonstrated that these DEGs were predominantly associated with key biological processes critical for oocyte competence, including oxidative stress response, energy metabolism, apoptosis regulation, and mitochondrial function (Figure 4B,C). Notably, differentially expressed genes included SIRT1, SIRT2, members of the caspase and SOD gene families, as well as the apoptosis regulators BAX and Bcl-2 (Figure 4D), indicating that POPC modulates transcriptional networks involved in redox balance, cell survival, and mitochondrial integrity.

These findings suggest that POPC enhances oocyte developmental potential by coordinately regulating gene expression programs linked to cellular homeostasis and metabolic fitness.

### 4.5. Regulation of Growth- and Apoptosis-Related Genes in Bovine Oocytes by POPC

To validate the differentially expressed genes identified through Smart-seq2 transcriptome sequencing, quantitative expression analysis was performed for key genes including SIRT1, SIRT2, SOD1, SOD2, Caspase-1, Caspase-3, Caspase-4, BCL2, and BAX.

**Figure 5:**
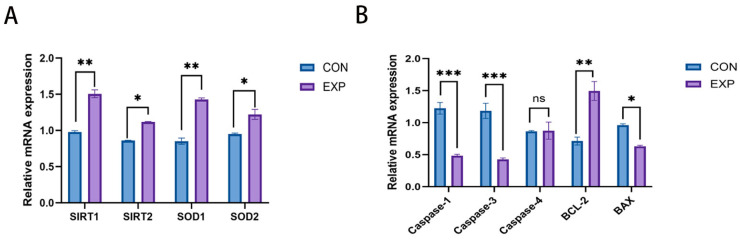
Analysis of relative expression levels of differentially expressed genes. Panel (**A**): Relative expression levels of antioxidant gene mRNAs. Panel (**B**): Relative expression levels of mRNA of apoptosis and pro-inflammatory related genes (ns: not significant, *p* > 0.05, *: *p* < 0.05, **: *p* < 0.01, ***: *p* < 0.001).

Results showed that antioxidant-related genes were significantly upregulated in the POPC-treated group compared to the control. Specifically, mRNA expression levels of SIRT1 and SOD1 were markedly increased (*p* < 0.01), while SIRT2 and SOD2 also exhibited significant upregulation (*p* < 0.05) (Figure 5A).

In apoptosis- and inflammation-associated genes, the pro-apoptotic gene BAX was significantly downregulated (*p* < 0.05), and the inflammatory mediators Caspase-1 and Caspase-3 showed pronounced reductions in expression (*p* < 0.001). Conversely, the anti-apoptotic gene BCL2 was significantly upregulated (*p* < 0.01). No significant difference was observed in Caspase-4 expression between groups (ns, *p* > 0.05) (Figure 5B). These findings confirm that POPC modulates critical molecular pathways governing oxidative stress resistance, cell survival, and inflammatory responses, thereby influencing oocyte developmental competence.

## 5. Discussion

In livestock production, oocyte quality represents a fundamental bottleneck restricting the efficiency of embryonic development. High-quality oocytes feature morphological integrity, homogeneous cytoplasmic texture, synchronized meiotic progression, and stable organelle function. Their maturation rate is strongly and positively correlated with subsequent embryonic cleavage rate, blastocyst formation rate, and pregnancy success following embryo transfer [15]. However, the in vitro culture environment differs substantially from the in vivo physiological milieu, frequently leading to oocyte aging. This aging manifests morphologically as a widened perivitelline space, polar body degeneration, zona pellucida hardening, and spindle disorganization [16], all of which compromise oocyte developmental competence and severely hinder the translational efficiency of IVM technologies [17].

To tackle this conundrum, emerging studies have focused on exogenous supplementation strategies to improve the oocyte maturation microenvironment and repair cellular dysfunction during IVM. Reported exogenous regulators encompass several categories of bioactive molecules. Antioxidants such as melatonin [18], glutathione [19], and hesperetin [20] alleviate OS damage by scavenging ROS and maintaining redox homeostasis. Mitochondrial protectants such as mogroside III [21], mogroside V [22], and coenzyme Q10 [23] preserve mitochondrial membrane potential, enhance oxidative phosphorylation efficiency, and ensure stable energy metabolism. Growth factors, by activating intracellular signaling cascades, promote cumulus cell expansion and oocyte cytoplasmic maturation [24]. Collectively, these studies demonstrate that the rational selection of exogenous additives and the optimization of their concentrations represent effective strategies for improving oocyte IVM quality and embryonic developmental potential, providing a theoretical and practical foundation for further exploring the regulatory roles of phospholipid molecules in the IVM system.

Based on this conceptual framework, this study investigated the effects of the phospholipid molecule POPC on bovine oocyte IVM by employing a gradient of concentrations. The study aimed to mitigate damage induced by in vitro culture, coordinate the regulation of OS and energy metabolism, and propose a novel functional supplementation strategy for improving oocyte quality and embryo developmental competence. The results demonstrated that 150 μmol/mL of POPC was the optimal concentration for exerting regulatory effects. At this concentration, the first polar body extrusion rate of bovine oocytes within COCs was significantly higher than that of the control group (*p* < 0.05). Cumulus expansion was well maintained, the perivitelline space remained within the ideal range (<3 μm), and the developmental rate of 2-4-cell embryos after IVF was significantly improved. In contrast, lower POPC concentrations were insufficient to reach the effective regulatory threshold, whereas higher concentrations triggered inflammatory responses that impaired embryonic development.

Mechanistically, 150 μmol/mL of POPC exerted multidimensional regulatory effects. At the redox homeostasis level, POPC markedly reduced intracellular ROS levels (*p* < 0.01) and increased GSH content (*p* < 0.001). Meanwhile, POPC upregulated antioxidant-related genes, including SIRT1, SIRT2, SOD1, and SOD2 (with SIRT1 and SOD1 showing highly significant differences, *p* < 0.01). These findings indicate that POPC alleviates OS by inhibiting ROS generation and enhancing antioxidant clearance capacity.

At the mitochondrial function and meiotic regulation levels, POPC significantly augmented mitochondrial content (*p* < 0.01), elevated the proportion of mitochondria with high membrane potential (ΔΨm) (*p* < 0.01), and reduced the proportion of depolarized mitochondria (*p* < 0.001), thereby ensuring sufficient energy supply. Moreover, the proportion of oocytes exhibiting normal spindle morphology increased markedly (*p* < 0.01), constituting the structural foundation for accurate chromosomal segregation. As the core of cellular energy metabolism, the functional integrity of mitochondria is a decisive factor for oocyte maturation and embryonic developmental competence.

During meiosis, spindle assembly and chromosome segregation are highly energy-dependent processes that require abundant ATP. Treatment with 150 μmol/mL of POPC supports energy homeostasis through two synergistic mechanisms: I: enhancement of mitochondrial activity, thereby increasing the total number of oxidative phosphorylation sites, and Ⅱ: inhibition of mitochondrial depolarization, which maintains respiratory chain efficiency and reduces ATP depletion. Proper spindle assembly and accurate chromosome segregation are critical for meiotic fidelity. Spindle abnormalities are the major contributor to aneuploid embryos. Notably, the proportion of oocytes with normal spindle morphology in the 150 μmol/mL POPC-treated group was significantly higher than that in the control group (*p* < 0.01). The spindles exhibited clear bipolarity, well-aligned microtubules, and no evidence of pole displacement or microtubule disorganization. This morphological optimization relies on dual mechanisms: first, sufficient ATP supply to power microtubule polymerization and molecular motor activity [25]; and second, potential modulation of microtubule-associated proteins (MAPs) by POPC [26], which prevents microtubule depolymerization and stabilizes spindle architecture. Improved spindle morphology can reduce the risk of chromosomal aneuploidy. Together with the observed enhancement in early embryonic developmental rates, it can be assumed that embryos from the POPC-treated group possess superior chromosomal stability.

At the transcriptomic and apoptotic regulatory levels, Smart-seq2 sequencing identified 630 differentially expressed genes (DEGs) with |log2FC| ≥ 1 and *q* < 0.05 [27]. These DEGs were mainly enriched in pathways related to OS, energy metabolism, apoptosis, and mitochondrial function. Pro-apoptotic genes BAX and Caspase-1/3 were notably downregulated (*p* < 0.001), whereas the anti-apoptotic gene BCL2 was significantly upregulated (*p* < 0.01), consistent with the activation of the “SIRT–antioxidant–antiapoptotic” signaling axis by POPC. POPC upregulated SIRT1 expression (*p* < 0.01), promoting its deacetylation-dependent antioxidant effects and inducing BCL2 expression (*p* < 0.01). The interaction of BCL2 with BAX inhibits the permeabilization of mitochondrial outer membranes, reduces cytochrome c release, and suppresses the activation of Caspase-3 and Caspase-1 (*p* < 0.001), thereby blocking endogenous apoptosis.

Although this study identified the optimal POPC concentration (150 μmol/mL) and elucidated key molecular mechanisms of POPC, specific target proteins and subsequent effects on embryo implantation and pregnancy outcomes remain to be investigated in future work. This study provides a scientific foundation for optimizing the in vitro culture system of bovine oocytes and enhancing the efficiency of assisted reproductive technologies in livestock.

In summary, this study demonstrates that 150 μmol/mL of POPC enhances bovine oocyte in vitro maturation and early embryonic development through multidimensional regulation. At the physiological level, it alleviates OS, improves mitochondrial function, and optimizes spindle morphology; at the molecular level, it activates the “SIRT–antioxidant–antiapoptotic” and energy metabolism pathways. As a natural phospholipid with low toxicity and high biosafety, POPC provides a simplified and biocompatible alternative to conventional culture systems containing complex chemical additives. This study thus lays a foundation for the standardization of in vitro maturation protocols, offers a novel strategy to mitigate oocyte aging and improve embryonic developmental competence, and contributes to genetic resource preservation and breeding efficiency in cattle. Moreover, the findings provide a valuable reference for oocyte culture optimization in other mammalian species, thereby advancing assisted reproductive technologies in livestock.

## 6. Conclusions

150 μM/mL of POPC is the optimal concentration: it can significantly increase the rate of first polar body extrusion and the cleavage rate of 2-4 cell embryos, reduce ROS, increase GSH, improve mitochondrial and spindle functions, and also regulate the activation of related pathways by controlling SIRT1/2, BCL-2, BAX, and Caspase-1/3 genes, providing a basis for optimizing assisted reproductive technologies in livestock.

## Figures and Tables

**Table 1:** Primer list.

Gene	Primer Sequences (5′-3′)	Size (bp)
GAPDH	F: ACGGGAAGCTCACTGGCATGG	227
	R: GCCAGCCCCAGCATCGAAG	
SIRT1	F: GCTGGGGTTTCTGTTTCTTGTGG	103
	R: TTGCTTGAGGATCTGGAAGGTCTG	
SIRT2	F: GCCGACCATCTGCCACTACTTC	92
	R: CTCGCTCCAGGGTGTCTATGTTC	
Caspase-1	F: GAGTGCTGAACCAGGAGGAGATG	156
	R: CTGCCAGGTGGGAGTCTTCTTC	
Caspase-3	F: GACAGACAGTGGTGCTGAGGATG	146
	R: TCTCACAAAGAGCCTGGATGAACC	
Caspase-4	F: TGCTTGGTGCTGTCATCTTGAGG	82
	R: GGGCATCTGGGCTTTAACATTTGG	
SOD1	F: CGGTGTTGCCATCGTGGATATTG	150
	R: CTTCCAGCGTTGCCAGTCTTTG	
SOD2	F: TCTGGACAAATCTGAGCCCTAACG	163
	R: TCCTTATTGAAGCCGAGCCAACC	
BAX	F: ATCATGGGCTGGACATTGGACTTC	154
	R: TGGTGAGCGAGGCGGTGAG	
BCL-2	F: CTGTGGATGACCGAGTACCTGAAC	149
	R: GCCAGACTGAGCAGTGCCTTC	

## Data Availability

The original contributions presented in this study are included in the article. Further inquiries can be directed to the corresponding author.
